# Absence of posture-dependent and posture-congruent memory effects on the recall of action sentences

**DOI:** 10.1371/journal.pone.0226297

**Published:** 2019-12-12

**Authors:** Antonio M. Díez-Álamo, Emiliano Díez, María A. Alonso, Angel Fernandez

**Affiliations:** 1 Departamento de Psicología Básica, Psicobiología y Metodología de las Ciencias del Comportamiento, Universidad de Salamanca, Salamanca, Spain; 2 Instituto Universitario de Integración en la Comunidad (INICO), Universidad de Salamanca, Salamanca, Spain; 3 Departamento de Psicología Cognitiva, Social y Organizacional, Universidad de La Laguna, Tenerife, Spain; 4 Instituto Universitario de Neurociencia (IUNE), Universidad de La Laguna, Tenerife, Spain; University of Sydney, AUSTRALIA

## Abstract

In two experiments with large samples of participants, we explored contextual memory effects associated with body posture, which was considered a physical and proprioceptive context and, therefore, potentially relevant to the encoding and retrieval of information. In Experiment 1 (*N* = 128), we studied the effect of context dependence on memory by manipulating the body posture adopted by the participants during the incidental encoding and subsequent recall of a series of action sentences not intrinsically associated with particular body postures (e.g., “*to put on a pair of glasses”*, *“to look at a postcard”*). Memory performance was not affected by context manipulation, as reflected by the absence of significant differences between remembering while in the posture adopted at study or in a different posture. Experiment 2 (*N* = 85) was designed to analyze context congruency memory effects, and for that purpose we manipulated the participants' body posture during the recall of sentences that described actions usually performed in body postures that were congruent or incongruent with the posture of the participants (e.g., recalling the sentence “*to travel by taxi*” while sitting or while standing). A content-neutral posture (lying) was used for the incidental encoding phase. Memory performance was not affected by contextual congruency at the time of recall, as evidenced by the lack of significant differences between recalling in a posture congruent with the content to be recalled and recalling in an alternative posture. Bayesian analyses supported the strength of null findings in the two experiments, adding to the evidence that, when taken together, the results in this study clearly failed to show contextual memory effects of body posture on the recall of action-related verbal statements.

## Introduction

Every single event or action in our lives inevitably takes place in a particular context, and context can affect the way things happen, the way we experience them, and the way we respond to them, with the potential to dramatically influence human behavior [1,2]. In the last decades, research in cognitive psychology has shown that context, in its different forms and definitions, plays a very important role in the operation of complex mental processes. For example, several studies have revealed that context influences perception (e.g., [3,4]), language comprehension (e.g., [5]), or emotion perception (e.g., [6]).

As far as memory is concerned, a widely studied phenomenon is the effect of external context on the encoding and retrieval of information, with the general finding that the recall of a certain material is better when the environmental encoding context coincides with the retrieval context; that is, it is easier to remember something when the learning context is reinstated at test. A notorious example of it is a classic experiment carried out by Godden and Baddeley [7] in which they studied this effect with a group of divers, who were presented with word lists underwater and out of the water, and were to subsequently recall them, either in the same context in which they had listened to them, or in the alternative context. The results showed that lists listened to underwater were better remembered in that same context than in the alternative one, and vice versa. Other authors have studied similar context-dependent memory effects manipulating overall changes in the experimental rooms [8,9], background visual displays [10], or even more specific features of the environment such as odors [11–13] or types of background music [14,15]. These findings are consistent with the encoding specificity principle, which states that recall is better when the conditions and memory cues at the time of retrieval match those present at encoding [16]. While there are reports questioning their replicability [17–19] or suggesting boundary conditions [20], most published studies have provided evidence that environmental context effects are overall reliable and present under a variety of conditions (for a review, see [21]).

Contextual effects on memory are not limited to the influence of the external environmental context, as they are also observable when the focus is put on the internal context, that is, when variables directly related to the person are involved. Here, the available evidence is even richer in scope, suggesting the operation of two varieties of contextual modulation of memory: dependency and congruency. Thus, context-dependent memory effects reflect the fact that recall is facilitated when the internal cognitive or emotional states during encoding and retrieval are coincident. An example of this is the fact that memory is helped by an encoding-retrieval match in the current cognitive context, that is, in thoughts, ideas, languages or motivational states [22,23]. Particularly interesting forms of internal dependency are shown in studies of mood and memory, which, despite some exceptions [24,25], demonstrate a clear tendency to recall information better when the mood state in which the person is during the retrieval phase matches that of the encoding phase (for a meta-analysis, see [26]). Furthermore, state-dependent memory effects have been found when the internal state is modulated by the consumption of drugs such as alcohol [27], tobacco [28] or other substances (for a review, see [29]), and also by natural changes in the physiological state [30]. A second class of contextual memory phenomena are known as context congruency effects, which refer to a facilitation in the recall of information when the content matches features of the internal context at the time of retrieval. Most demonstrations of this variety of contextual effects on episodic memory come from studies manipulating the emotional valence of studied materials to make it either congruent or not congruent with the particular mood state of an individual at the time of retrieval [31,32].

In the light of the relatively vast amount of evidence supporting the importance of context effects on memory, it is surprising that very few studies have considered body posture as a relevant factor for the processing and retrieval of information. A clear antecedent is a study carried out by Helen J. Reed in 1931, who asked participants to memorize word lists and manipulated, among other variables, their body posture during the learning phase and during the recall test. Specifically, half of the subjects studied the words while seated and the other half in a standing posture. During a recall test, that took place 24 hours later, half of the participants in each group adopted the same body posture and the other half adopted the alternative posture. Reed [33], in what can be regarded as an early attempt to analyze context-dependent memory, reported unreliable differences in recall scores under the four possible postural conditions. By contrast, Rand and Wapner [34] conducted a similar experiment in which nonsense-syllable learning and 15-min-delayed relearning were assessed under conditions in which the body postures of participants (standing and supine) were the same or different in the learning and in the relearning phases. In this case, the authors found evidence of significantly greater savings in relearning when the posture matched the one adopted during initial learning. Very recently, Hammond, Murphy, Silverman, Bernas and Nardi [35] conducted a related study in which the participants encoded the location of objects either when they were walking or when they were standing. In a later recall test, they were to remember the location of the objects while either walking or standing. Their results did not support a body-status effect on object-location memory. Regarding congruency effects, Riskind [36] demonstrated that smiling and adopting an upright posture facilitated the retrieval of pleasant experiences, while a sad facial expression and a stooped posture facilitated the retrieval of unpleasant experiences. Note, however, that the interpretation of this latter result in terms of body postures is further complicated by the fact that this manipulation involves a combination of posture and facial expressions.

Recent developments in theoretical approaches to cognitive neuroscience have led to a renewed interest in the possibility that body posture can be a significant context for information processing and memory. Perhaps, the most notable argument comes from the embodied cognition theoretical framework, an approach that claims that all psychological processes depend on the bodily and neural systems related to perception, action, and emotion [37,38]. In this sense, the concept of *simulation* is employed to suggest that these systems are used to reenact our experiences in the world, which is the basis of the functioning of language comprehension, memory and other cognitive processes (e.g., [39,40]). Importantly, this perspective posits that cognition is not located exclusively in the brain, or in other words, that it is not independent of the body. On the contrary, cognition depends on both the brain and the body, in interaction with the physical and social environment [41], especially when considering that our body is the most direct interface with the environment. Thus, although body posture could be considered as an external or internal context, our approach, inspired by embodied cognition, is to consider it internal, due to the direct involvement that the body has in the cognitive mechanisms that underlie perception, action and emotion.

The literature on embodied cognition contains numerous examples of studies that show how body posture can affect diverse psychological aspects such as mood and thoughts [42], self-related attitudes [43], quantitative estimates [44], physiological reactions to appetitive emotive stimuli [45], cognitive dissonance and rationalization [46], and approach motivation [47]. Of greater interest for our purposes, some embodiment-oriented researchers have studied how body posture can constitute a relevant processing context, capable of affecting memory.

For example, extending the results of Riskind [36], described above, Dijkstra, Kaschak, and Zwaan [48] demonstrated that adopting a certain body posture facilitates the retrieval and posterior retention of autobiographical memories congruent with such body posture in younger and older adults. In another study, Michalak, Mischnat, and Teismann [49] found that depressed patients sitting in a slumped posture recalled more negative words from a studied set of positive (e.g., “beauty”) and negative (e.g., “exhaustion”) words, whereas depressed patients sitting in an upright posture showed more balanced levels of recall of positive and negative words. Similarly, Michalak, Rohde, and Troje [50] found that manipulating the walking pattern of a group of students to resemble either a depressive gait or a happy gait biased the recall of a series of emotionally loaded words, revealing a congruence effect (see also [51]). Finally, Dutriaux and collaborators [52,53] found that adopting a posture that interferes with manual action (hands behind one's back) reduced the recall of pictures and words denoting manipulable objects as opposed to nonmanipulable ones and decreased memory for words referring to manipulable objects when they were associated with action verbs at encoding. Thus, it can be argued that the recall of material is primed when the bodily and neural systems related to perception, action, and emotion are activated in a manner that is congruent and integratible with the content of such material (see [54]), for example, by adopting a specific body posture, as memory depends on simulation.

The present study was designed to further investigate contextual memory effects from the perspective of embodied cognition, contributing to the extant literature and, importantly, considering body posture as a relevant and significant context for the processing and retrieval of action-related information acquired under incidental learning circumstances. Thus, the state of the bodily and neural sensorimotor systems during the encoding and retrieval of information is expected to generate relevant memory cues and to modulate simulation processes, yielding contextual memory effects. For this purpose, we decided to use a double procedural approach. On the one hand, Experiment 1 was aimed at exploring potential posture-dependent memory effects, and on the other hand, Experiment 2 was designed to analyze posture-congruent memory effects. The analysis of both types of contextual memory effects in the same study allows for the joint consideration of two related phenomena under very similar conditions with regard to design, materials, and participants, which is a singularity of the present research. Likewise, the use of a set of standardized well-characterized action sentences (see below) allows for good experimental control of the relevant variables, contributing to the clarity of the results. In addition, the use of action-related sentences as to-be-remembered material, already shown to be useful for studying some of the effects related to embodied cognition, such as enactment effects (see a summary in [55]), may be particularly useful for the exploration of bodily effects on memory retrieval. Finally, the stimuli used in the present study had a neutral emotional value and the body postures used were not intended to elicit mood states. In this way, we aimed to study the contextual memory effects associated with body posture in the absence of affective or emotional factors, which have already been shown to be relevant for contextual memory effects, as mentioned above. Therefore, we consider that, by focusing solely on the effects of posture manipulations on memory for emotionally neutral materials, the present research has the potential to significantly contribute to the study of body-related contextual memory effects.

## Experiment 1

The objective of this experiment was to study the effect of context dependence on memory for action statements, taking the body posture adopted at encoding and retrieval as an internal context of potential relevance for the processing of information. More specifically, two differentiated body postures were used, namely sitting and standing, the assumption being that a list of sentences would be better recalled when the body posture adopted at retrieval matched the posture adopted at encoding, compared to a mismatch situation. The experiment consisted of three differentiated phases within an incidental-learning paradigm: a first encoding phase, in which the participants listened to 24 sentences while making familiarity judgments about the actions described in them, a 10-min retention interval during which a distracting task was administered, and a final retrieval phase. We chose to use a free recall test, since it has been reported to be more adequate to find context dependence effects than recognition tests [9,29]. Participants were randomly assigned to one of the two body postures in both phases of the experiment, resulting in four possible groups. Therefore, the experimental design was a 2 x 2. Body posture at encoding and at retrieval, either sitting or standing, were the independent variables, both between subjects, and the dependent variable was the proportion of correctly recalled sentences.

In addition, given the aforementioned mixed results in the extant literature on contextual memory effects, we collected participants' information regarding two variables with potential implications for the studied effects to be used as covariates. One was the number of hours of sleep the night before the experiment, and the other was the exercise habits of the participants (specifically, the number of times they exercised per week). The inclusion of the number of hours of *sleep* as a covariate is based on a study by Muehlhan, Marxen, Landsiedel, Malberg and Zaunseder [56], which showed that the quality of sleep affected the performance of a group of participants in an N-back working memory task when the task was carried out in a supine body posture, but not when in a sitting posture. Although our measurement only indicates the number of hours of sleep and not the quality of sleep, we decided to use it as a covariate because of its potential predictive value, since sleep deprivation itself has also been shown to affect memory [57]. The covariate *exercise* was included not only due to the fact that physical activity has been shown to be beneficial for memory [58,59], but also because we contemplated the possibility that exercising regularly could enhance one's body awareness, which would make body-related memory cues more salient, modulating postural-dependent memory effects.

The purpose of this experiment was to shed light on the contradictory data reported by Reed [33], who found no significant dependence effects, and by Rand and Wapner [34], who found evidence of significantly greater savings in relearning scores when the learn and relearn postures matched, as explained above.

### Methods

#### Participants

An *a priori* power analysis with G*Power [60] indicated that, in order to achieve 80% power to detect a medium effect size (Cohen's *f* = .25, *α* = .05) with a between-subjects design, a total sample of 128 participants would be required.

A total of 135 undergraduate students from the University of Salamanca (Spain) participated in the experiment in exchange for course credits. Participation was voluntary and the students signed an informed consent form. The study was approved by the Ethics Committee of the University of Salamanca and was conducted in accordance with the principles of the Declaration of Helsinki [61]. A questionnaire was used to collect participants' demographic data and information on the number of hours they had slept the night before the experiment and on their exercise habits. To control for potential effects of fatigue and lack of sleep on memory (see [57]), students who reported less than four hours of sleep the night before the session were excluded from the experiment. No previous information was given to the participants about the hypothesis of the experiment, nor about the existence of a memory test at the end of the experiment. Three participants anticipated the existence of this test, and four failed to follow the instructions of the experiment properly. Although the data obtained from these seven individuals were collected, they were not included in the analyses. Thus, the final sample included 128 participants (100 female, 28 male), all of them native Spanish speakers, with a mean age of 19.3 years (*SD* = 3.0; range = 17–43 years).

#### Stimuli

A list of 24 three-word sentences which described simple actions (see S1 Appendix) was used as to-be-remembered material. The sentences were neutral in relation to body posture, since the actions described in them (e.g., “*to put on a pair of glasses”*, *“to look at a postcard”*) are not usually carried out in a characteristic or preferential way in any of the body postures used in this experiment (sitting or standing). The sentences were obtained from a previous normative study [55] in which familiarity, emotionality, motor activity, memorability and vividness of visual imagery ratings were collected for 536 action phrases, using 7-point scales. Twenty-four sentences were chosen, ensuring the inclusion of sentences throughout the range of the familiarity variable (*M* = 4.5, range = 1.5–6.8). In addition, all sentences had similar average scores in memorability (*M* = 4.0, range = 3.2–5.0). Also, the length of the sentences was controlled, so that they had a similar number of characters (*M* = 16.8, range = 13–20). Finally, to avoid unwanted interferences in recall, the nouns and verbs contained in each of the sentences were not repeated in other sentences of the list. The sentences were digitized into an audio format using custom-made Text-To-Speech software, selecting a Spanish female voice.

#### Procedure

Participants were individually run. In a first room, they filled in a data sheet, read and signed the informed consent form, and read the instructions for the experiment on a computer screen. Next, they were led to another room where all the phases of the experiment took place. The first task was to evaluate the familiarity of the actions described in each of the 24 sentences, randomly presented, using a 7-point scale, where 1 represented actions with low frequency of occurrence and 7 represented actions with high frequency of occurrence. Specifically, the instruction was to assess how familiar or frequent each action was according to their own experience. This activity was an orienting task aimed at ensuring semantic processing during the incidental learning of the sentences. To facilitate the familiarity assessment and to encourage simulation of the content of the sentences, participants were asked to imagine themselves performing each action, and they were informed that they did not need to respond quickly, since it was not a speed test. The sentences were presented auditorily by means of headphones and the participants used an auxiliary USB numeric keypad to deliver their response to each sentence, within a 12-second time window. Half of the participants performed this task while sitting on a chair at a table on which the numeric keypad was located, and the other half did so while standing upright in front of a 140 cm high wooden stand, where the numeric keypad was placed. The assignment of participants to each of these postures was random, with the restriction of maintaining the same proportion of male and female participants across conditions. Immediately after the end of this task, participants were led to a stretcher where they were asked to lie down on their back. The table, the wooden stand and the stretcher were all in the same room, separated 1.5 m from each other. The head end of the stretcher was reclined at an angle of approximately 20 degrees, so that the participant's head and shoulders were slightly raised to facilitate the use of the numeric keypad. The participant's legs were fully stretched horizontally. This neutral body posture was used during the entire retention interval, in which participants were auditorily presented with a series of mathematical operations, consisting of the addition and / or subtraction of three numbers from 1 to 9, to which they had to answer using the auxiliary numeric keypad. They received immediate feedback on the accuracy of their answers. In this case, a male voice was used for the locution of the operations and feedback messages, in order to differentiate it as much as possible from the female voice used in the presentation of the sentences. This highly demanding task was designed to prevent any type of rehearsal of the sentences and had a duration of 10 minutes, which constituted the retention interval. Immediately after this, the participants stood up and were led to the place where the unexpected memory test took place, which consisted in a standard paper-and-pen free-recall test. Half of the participants took the test sitting at the table and the other half did so while standing in front of the wooden stand. Participants were randomly assigned to one of the two postures, with the restriction that the four resulting groups be equally completed and that the proportion of male and female participants across conditions be the same. Participants were instructed to try to remember and write as many sentences of those heard in the first task as possible, in any order, and as accurately as possible. The pace to perform the memory test was free, with a limit of 10 minutes. The total duration of the experiment was approximately 25 minutes. After the experiment was finished, the participants were asked if they had anticipated the final memory test, in order to discard the data of those who had. They were also asked not to comment on the experiment with other students.

### Results and discussion

Two different systems were used to score the participants' responses in the free recall test: literal scoring and flexible scoring. The literal scoring system implied that the recall of a sentence was considered correct only when the sentence was recalled exactly as it was presented in the first phase of the experiment. That is, any change in the original sentence made it into an incorrect response. Conversely, the flexible scoring system allowed small variations or changes in the sentences, such as the use of synonyms, as long as the essential content of the sentences remained unchanged (e.g., “to push a button” was taken as a correct recall response for the original “to press a button”). Table 1 lists the mean proportions of correctly recalled sentences according to both scoring systems, in each condition of the experimental design.

**Table 1:** Experiment 1. Mean proportion (M) and standard deviation (SD) of correctly recalled sentences in each condition of the design, according to a literal or flexible scoring.

		Literal scoring		Flexible scoring		
Posture at encoding	Posture at retrieval	*M*	*SD*	*M*	*SD*	*N*
standing	standing	0.25	0.12	0.40	0.14	32
	sitting	0.25	0.09	0.40	0.14	32
sitting	standing	0.23	0.10	0.37	0.11	32
	sitting	0.26	0.12	0.39	0.14	32

A 2 (body posture at encoding: sitting *vs*. standing) x 2 (body posture at retrieval: sitting *vs*. standing) analysis of variance (ANOVA) on the proportion of correctly recalled sentences, according to the literal scoring system, showed no significant main effects due to either body posture at encoding [*F*(1,124) = .01, *p* = .92, *η*^*2*^_*p*_ < .001] or body posture at retrieval [*F*(1,124) = .74, *p* = .39, *η*^*2*^_*p*_ = .01]. In addition, the interaction between these two factors was not significant [*F*(1,124) = .52, *p* = .47, *η*^*2*^_*p*_ < .01]. Subsequently, a default priors Bayes factor analysis performed with JASP software [62] showed that the data were 63.2 times more likely to occur under the model with no interaction (BF_01_ = 63.2). A parallel ANOVA was conducted on the proportion of correctly recalled sentences, according to the flexible scoring system. Again, no significant main effects due to either body posture at encoding [*F*(1,124) = .65, *p* = .42, *η*^*2*^_*p*_ = .01] or at retrieval [*F*(1,124) = .04, *p* = .85, *η*^*2*^_*p*_ < .001] were found, and the interaction was not significant [*F*(1,124) = .09, *p* = .76, *η*^*2*^_*p*_ < .01; BF_01_ = 77.60].

In a second step, analyses of covariance (ANCOVAs) were used to test for the covariates *sleep* (number of hours of sleep the night before the experiment) and *exercise* (number of times per week in which some kind of physical exercise was practiced). However, the inclusion of these two covariates did not alter the results substantially, as there were no significant main effects or interactions involving the body posture at encoding and at retrieval factors, neither in the literal nor in the flexible scoring (all *F*s < 1, *p*s > .40).

The absence of significant posture-dependent memory effects in our results is in contradiction to the same-context advantage reported by Rand and Wapner [34] using a similar postural manipulation. In their study, relearning of nonsense syllables was aided by reinstating the learning posture at recall, although the effect was relatively small, limited to the early stages of recall, and might have to do with the fact that the to-be-learned material was devoid of meaning and new to the participants. The results of the current experiment are, however, in line with the null findings reported by Reed [33] with meaningful material (words) and a more comparable memory test. Note that the study reported here is closer in methodology to Reed's [33] study than to Rand and Wapner's [34] study, and more powerful in terms of the number of participants than both earlier studies (128 in the present experiment versus 78 in Reed’s study and 32 in Rand and Wapner’s study). In the light of the available evidence, which is admittedly scarce, a reasonable conclusion is, therefore, that body posture manipulations only produce context dependence effects under certain circumstances, and are hardly generalizable to other procedural conditions, such as those used in the present experiment, possibly because body-related memory cues are not always strong or salient enough to modulate the interaction between learning and recall.

## Experiment 2

The purpose of this experiment was to further examine contextual manipulations on memory for actions by analyzing potential posture-congruent memory effects, with the expectation that, if body posture functions as a contextual modulator, information describing actions that are functionally congruent with the body posture adopted at retrieval would be more accessible than non-congruent information. To this end, we used a set of sentences describing actions that are usually performed either sitting down (e.g., *“to travel by taxi”*) or standing up (e.g., *“to open a mailbox”*). Importantly, we manipulated the body posture adopted by the participants during the retrieval phase. Half of the participants, randomly selected, did the memory test while sitting and the other half while standing. Thus, within the to-be-remembered set, there were sentences that were congruent and sentences that were incongruent with the body posture adopted by the participants at retrieval. To avoid confounding effects, all participants were in a neutral lying posture during the encoding phase. As with Experiment 1, this experiment consisted of three phases: a first incidental encoding phase, in which the participants made familiarity judgments on 24 action sentences, a 10-min retention interval during which a math task was administered, and a final unexpected free recall test. This experiment had a 2 x 2 mixed factorial design. The body posture at retrieval was a between-subjects factor, the sentence type was a within-subjects factor and the proportion of correctly recalled sentences was the dependent variable. In addition, as with Experiment 1, we explored the potential impact of the *sleep* and *exercise* covariates.

We anticipated that this experiment, despite the existing methodological differences, could provide evidence consistent with the results of the studies by Riskind [36] and Dijkstra et al. [48], which have shown significant effects of postural congruence on autobiographical memory.

### Methods

#### Participants

A total of 91 undergraduate students from the University of Salamanca (Spain) participated voluntarily in the experiment in exchange for course credits, and all participants signed an informed consent form. The study was approved by the Ethics Committee of the University of Salamanca and is in accordance with the principles of the Declaration of Helsinki [61]. As with Experiment 1, to take part in the experiment, participants should have slept at least four hours the night before. There was no overlap with the participants in Experiment 1, and no previous information was given to the participants about the hypothesis of the experiment, nor about the existence of a final memory test. All the data obtained from six participants who did not follow the instructions of the experiment properly were discarded. When questioned at the end of the session, none of the participants stated to have anticipated the final memory test. The final sample included 85 participants (67 female, 18 male), all of them native Spanish speakers, with a mean age of 18.2 years (*SD* = 1.4; range = 17–26 years).

#### Stimuli

A total of 24 action sentences, between 3 and 5 words long, were used (see S1 Appendix). Half of them described actions that are usually carried out while sitting (e.g., *“to travel by taxi”*), and the other half described actions that are usually performed while standing (e.g., *“to open a mailbox”*). The sentences, obtained from a previous normative study [55], were well distributed throughout the range of the familiarity variable in both types of sentences (sitting sentences: *M* = 3.6, range = 1.2–6.0; standing sentences: *M* = 4.4, range = 1.5–6.2). In addition, the two groups of sentences were similar in memorability (sitting sentences: *M* = 4.6, range = 3.3–5.8; standing sentences: *M* = 4.3, range = 3.8–5.2) and length (sitting sentences: *M* = 17.7 characters, range = 11–26; standing sentences: *M* = 17.8 characters, range = 14–24). As in Experiment 1, the nouns and verbs contained in each of the sentences were not repeated in the rest of the stimuli set. The sentences were digitized into an audio format using custom-made Text-To-Speech software, selecting a Spanish female voice.

#### Procedure

Participants were individually run. The procedure and instructions were the same as in Experiment 1, except for the following differences: all the participants performed the incidental encoding phase lying on the stretcher, carrying out the familiarity judgments on the 24 sentences, randomly presented through headphones, by means of the auxiliary USB numeric keypad; the mathematical operations task, which constitutes the 10-min retention interval, was also performed in this neutral body posture; and, finally, half of the participants did the unexpected paper-and-pen free recall test sitting at the table and the other half did so while standing upright in front of the wooden stand. Participants were randomly assigned to one of these body postures, with the restriction that both groups be equally completed, and that the proportion of male and female participants be the same in both conditions. The pace to perform the memory test was free, with a limit of 10 minutes. The total duration of the experiment was approximately 25 minutes. Upon completion of the experiment, the participants were asked if they had anticipated the final memory test, and they were asked not to comment on the experiment with other students.

### Results and discussion

As in Experiment 1, both the literal and the flexible scoring systems were used to validate participants' responses in the free recall test. Mean proportions of correctly recalled sentences according to both scoring systems, in each condition of the experimental design, are shown in Table 2.

**Table 2:** Experiment 2. Mean proportion (M) and standard deviation (SD) of correctly recalled sentences in each condition of the design, according to a literal or flexible scoring.

		Literal scoring		Flexible scoring		
Body posture at retrieval	Sentence type	*M*	*SD*	*M*	*SD*	*N*
sitting	sitting action	0.27	0.15	0.47	0.15	43
	standing action	0.28	0.13	0.45	0.15	43
standing	sitting action	0.30	0.12	0.52	0.16	42
	standing action	0.21	0.14	0.42	0.16	42

**Fig 1:**
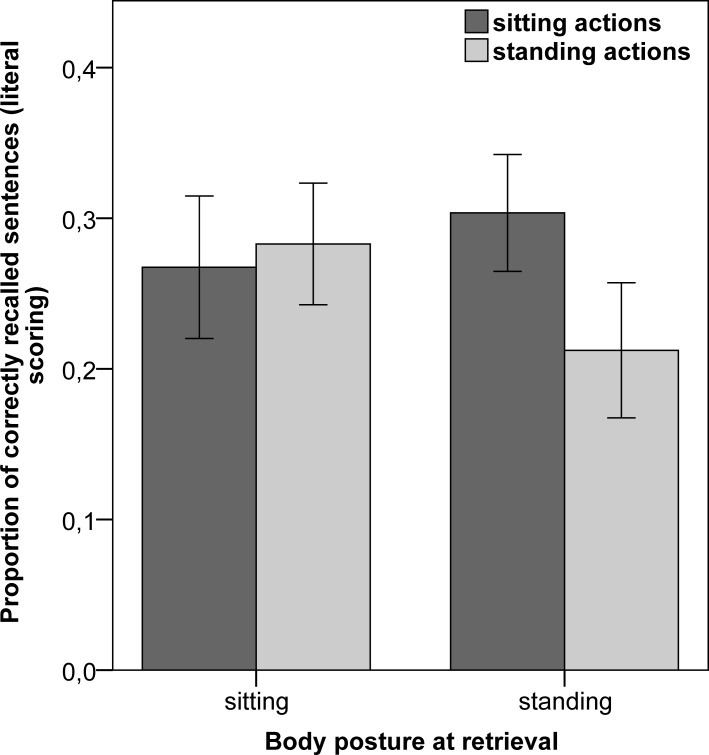
Proportion of correctly recalled sentences (literal scoring) as a function of sentence type and body posture at retrieval. Error bars represent a 95% confidence interval (CI).

A 2 (sentence type: sitting action *vs*. standing action) x 2 (body posture at retrieval: sitting *vs*. standing) mixed ANOVA on the proportion of correctly recalled sentences, according to the literal scoring system, revealed a significant main effect of sentence type [*F*(1,83) = 4.92, *p* = .03, *η*^*2*^_*p*_ = .06], indicating that the actions likely to be performed while sitting were recalled to a greater extent (*M* = .29) than the standing actions (*M* = .25). In contrast, the body posture adopted at retrieval did not produce a significant main effect [*F*(1,83) = .48, *p* = .49, *η*^*2*^_*p*_ = .01]. The interaction between the two factors proved significant [*F*(1,83) = 9.78, *p* < .01, *η*^*2*^_*p*_ = .11; BF_01_ = 0.196], with a high observed power (89%) to detect the interaction, calculated with G*Power [60]. As can be seen in Fig 1, there were no differences in the proportion of correct recall between sitting and standing action sentences in the group of participants who were sitting at retrieval, whereas those who were standing at retrieval recalled a smaller proportion of standing actions (*M* = .21) compared to sitting actions (*M* = .30).

Another ANOVA was conducted on the proportion of correctly recalled sentences, according to the flexible scoring system. Again, the results showed that participants recalled more sitting actions (*M* = .49) than standing actions (*M* = .43) [*F*(1,83) = 8.79, *p* < .01, *η*^*2*^_*p*_ = .10], and that the body posture adopted at retrieval did not produce a significant main effect [*F*(1,83) = .07, *p* = .80, *η*^*2*^_*p*_ < .01]. However, in this case, the interaction between sentence type and body posture at retrieval did not reach statistical significance [*F*(1,83) = 3.60, *p* = .06, *η*^*2*^_*p*_ = .04; BF_01_ = 0.513].

In a second step, ANCOVAs were performed including the covariates *sleep* and *exercise*. The analyses yielded no significant main effects of sentence type or body posture at retrieval, neither in the literal nor in the flexible scoring (all *F*s < 1.20; *p*s > .25). Regarding the interaction between sentence type and body posture at retrieval, it was significant for the literal scoring system [*F*(1,81) = 9.15, *p* < .01, *η*^*2*^_*p*_ = .10], reflecting a similar pattern of results to that obtained in the previous ANOVA (literal scoring), but, again, it did not reach statistical significance in the case of the flexible scoring system [*F*(1,81) = 3.16, *p* = .08, *η*^*2*^_*p*_ = .04].

These results do not support the existence of posture-congruent memory effects. On the contrary, the significant interaction observed in the proportion of correctly recalled sentences under the literal scoring system reflects an effect in the opposite direction, since the participants who adopted a standing posture at retrieval recalled fewer congruent than incongruent actions. This conclusion was supported by a Bayes factor analysis (null/alternative) that yielded substantial evidence (BF_01_ = 0.196) in favor of the alternative hypothesis, that is, the data are 5.1 times more likely to take place under the model including this interaction, rather than under the model without it (see [63]). Also, the observed power to detect the interaction found was high (89%). A tentative explanation of these results could be that the sitting posture represents a natural and habitual recall/test situation for a sample of university students, making them potentially less aware of their body posture, more oblivious to the body-related memory cues and their relation to the action sentences, and more likely to use alternative cues or strategies to retrieve the sentences. This would have prevented the observation of congruence effects in the group of participants who were sitting at retrieval. Adopting a standing posture at retrieval, however, could have provoked a novel and unnatural situation for the participants (as noted by Reed, [33]). Such situation would require a certain degree of extra motor processing that could interfere with the motor simulation potentially involved in motor-content retrieval in case the participants were not able to integrate their own physical posture into the simulation of the content of the sentences (see Kaschak et al. [54] for a description of factors that interact in determining when a match/mismatch advantage is expected). As a result, the recall of sentences describing standing actions could have been impaired in the standing retrieval condition. It should be noticed, though, that because the experiment was not originally conceived to be a direct test of these assumptions, this interpretation should be considered with caution and submitted to further empirical scrutiny.

The overall results of this experiment, thus, failed to show a reliable congruency effect of body posture on the recall of sentences describing actions typically performed in the manipulated postures. Although conceived and designed bearing resemblance to prior studies exploring posture-congruent effects on declarative memory [36,48], the current experiment presented characteristics that, in our view, make it better suited to examine the effects of body posture on the recall of congruent information. Earlier studies were focused on the recall of personal experiences that took place long before the experiment, which implies lack of control over factors such as episode-encoding conditions, possibilities of subsequent rehearsal, the centrality of episodes in the participants' lives, and even the authenticity of the memories. In contrast, the present study is based on a well-controlled set of action phrases that participants encoded incidentally under standardized conditions at the beginning of the experiment. Another significant difference is that, unlike previous studies [36,49,50], our to-be-remembered material does not possess a marked emotional valence, nor are the chosen body postures likely to elicit a particularly marked emotional mood. Finally, and unlike the study by Michalak et al. [49], participants were drawn from a standard college pool, and not selected from a clinically defined population. Consequently, the current experiment focused on analyzing the effects of postural congruence on memory, intentionally eliminating from the design a number of potentially confounding factors related to the emotional valence of the stimuli, the emotional significance of body postures, and the emotional state of the participants. In doing so, it is likely to present optimized conditions for the detection of uncontaminated posture-congruent memory effects.

## General discussion

In the present study, we explored contextual memory effects associated with body posture. From the perspective of embodied cognition and on the basis of a review of the evidence for both external and internal context effects on memory, body posture could be expected to provide a relevant and significant context for information processing and memory. Because the literature presents some relevant cases in which the evidence for contextual effects is not reliable, two independent experiments were designed. Experiment 1 was aimed at studying posture-dependent memory effects, and experiment 2 was designed to analyze posture-congruent memory effects. As described above, neither of the two experiments provided reliable evidence of such effects on the recall of action-related verbal material.

Given these null effects, it could be argued that, despite the human body being a permanent presence, accompanying “the knowledge of whatever else we know” ([64], p.241), body-related memory cues are not relevant enough to modulate encoding and recall. This conclusion stands in contradiction to the significant memory effects associated with postural contexts reported by several other researchers [34,36,48–50], although it should be noted that, as mentioned above, some of the cited studies manipulated not only body postures, but also emotional or affective factors.

There seems to be no lack of statistical power in either of the experiments, as the size of the samples used allowed us to achieve a high power and exceeded the number of participants in the most paradigmatic and widely cited studies, such as Godden and Baddeley [7] with *N* = 18 (Exp. 1), or Smith et al. [9] with *N* = 24 (Exp. 2). Moreover, Bayesian analyses supported the strength of null findings in the two experiments. A plausible explanation for the substantiated absence of contextual effects in the present study is that the body postures used in the experiments simply did not represent a salient enough context and, consequently, the participants did not perceive the relation between the postural context and the action sentences processed in the experiments as a relevant aspect of the task. In reporting their null effects of environmental context manipulations, Fernandez and Glenberg [18] suggested that an experiment, as a whole, may constitute in itself a context that does not change from the participant's point of view throughout its full duration (study–retention interval–test), despite the fact that certain (postural) changes are eventually introduced. Therefore, the encoding and recall of the sentences in both experiments could have been unaffected by postural manipulations that were perhaps not salient enough for the participants. In that sense, it is possible that the use of body manipulations involving more distinct positional states (e.g., sitting *vs*. walking) could lead to differential context effects. However, in such cases, the control of experimental conditions could be compromised by effects other than postural variations. For example, it has been shown that exercising implicates a heightened level of arousal, leading to the possibility of such feature being encoded as part of the episodic representation and, subsequently, being used as an effective retrieval cue [65], in addition or instead of pure postural retrieval cues.

Following Reed [33], it could also be argued that the absence of context effects could be related to the lack of naturalness of the experimental conditions. The forced adoption of specific body postures, either during the encoding phase, the retention interval or the retrieval phase, could have generated novel and artificial situations for the participants that differ from the usual contexts of information processing, causing a dissociation between induced body conditions and the acquisition of verbal information. Indeed, this line of reasoning would be consistent with Fernandez and Glenberg [18], who suggested that establishing a relation between the context and the information to be retrieved could be a fundamental factor for the observation of context effects, since it would provide coherence, cohesion and even causality to the event within its context, as it is likely to occur in a variety of natural situations.

Another plausible explanation could be derived from the outshining hypothesis [66,67], that claims that retrieval cues from the context are not always crucial for mnemonic processing, but are used only when there are no better alternative cues available. According to this idea, it could be argued that the participants did not use the physical body-related cues during the tasks, making use of alternative retrieval cues instead, with their recall being eventually influenced by unknown variables and mental processes. Alternatively, as maintained by Bjork and Richardson-Klavehn [68], the participants could have been able to mentally reinstate the encoding context independently of the body posture adopted at retrieval, which would also hinder the observation of context effects.

Finally, the fact that participants had to write down the sentences during the free recall test might have produced motor interference in the simulation of a number of sentences that contained a certain degree of manual action, making contextual effects more difficult to develop. Although this interpretation would require additional research to be verified, it would be in line with recent studies that showed that interfering with manual action by keeping one's hands behind one's back decreases the memory for information related to manual action [52,53].

All these, and other potentially relevant alternative accounts of the data, present arguments that are worth considering and could be enlightening. However, given the characteristics of the experiments conducted in the present study, the fact is that the reported results do not allow for the possibility of fair testing among interpretations or even for the formulation of substantiated *a posteriori* predictions. Therefore, and with the data at hand, the most sensible conclusions at this point are that posture-based dependency and congruency effects are far from being universally observable, and that further work is needed on which could be crucial induction and modulating conditions. From a more general perspective, the reported negative findings constitute an additional piece of evidence reflecting the lack of generality of context dependency effects on the recall of verbal information, already documented by a number of previous studies.

In closing, it should be noted that the two experiments reported here were conducted to address an important psychological issue, they focused on relevant questions identified through logical steps from available theories and data, they made use of adequate designs, stimuli and manipulations, they were conducted on sufficiently large samples, and they provided results that were clearly against the rejection of the null hypotheses regarding posture-dependent and posture-congruent memory effects. Because of their nature, the negative results presented here are not likely to make a particularly great contribution to the development of theoretical accounts of memory functioning. Nonetheless, the importance of accumulating evidence based on empirical findings should not be underscored, particularly in areas such as context-related memory, where there is a documented history of contradictory results. Additionally, and as recently pointed out by Wälti et al. [19], applied psychologists (in the fields of education, forensics or clinical psychology) usually assume that the facilitatory effects of context reinstatement are a reliable and generalizable phenomenon, and have used the concept when interpreting results and designing interventions, which constitutes another reason for the effort in considering all sorts of validly obtained empirical evidence. Thus, although body posture manipulation may have potential applications in education [69] and clinical psychology [49,50], the absence of posture-dependent and posture-congruent memory effects in the present study shows the need to further testing of the effectiveness of educational and clinical interventions that are based on postural context manipulations.

We, therefore, believe that the findings presented in this study represent an informative and important contribution to the study of posture-related contextual memory effects, and can constitute a valid step in the search for conceptual and direct replications of this kind of empirical effects. Although more research is still needed to clarify the circumstances under which the aforementioned effects can be observed, the currently presented evidence will be especially useful for the development of meta-analytic studies and long-term projects on this subject. In addition, as recently posited by Berg [70], conceptual replications, such as those carried out in the present study, allow us to test the robustness of the studied effects, which can also inform about their applicability for practical purposes. Finally, it is our position that future studies on memory and postural context could greatly benefit from the implementation of procedures that: 1) optimize the naturalness and ecological validity of the body postures adopted by participants within the experimental situation; 2) highlight the salience of body-related memory cues during the experiment, for example by using less habitual body postures, and try to attenuate the effect of alternative memory cues or retrieval strategies; and 3) facilitate that participants perceive the relevance of the postural context for the memory task, favoring the link between the bodily context and the information to be retrieved. In these ways, we consider that it would be possible to better analyze contextual memory effects associated with body posture, with results expected to be of relevance to this particular issue, and also relevant for the general domain of contextual effects on memory.

## Supporting information

S1 Appendix Sentences used in the experiments. (PDF)
