# Supplementary material for: Absence of posture-dependent and posture-congruent memory effects on the recall of action sentences
Source: PLoS One. 2019 Dec 12;14(12):e0226297. doi: 10.1371/journal.pone.0226297 (PMC6907800; doi:10.1371/journal.pone.0226297)
Supplement: S1 Appendix — (PDF) [file pone.0226297.s001.pdf]

S1 Appendix: Sentences used in the experiments.

Experiment 1

Spanish sentence	English translation
apretar los dientes	to clench one's teeth
arrugar un pañuelo	to crumple a handkerchief
cerrar un bolso	to close a purse
comer una manzana	to eat an apple
cruzar los dedos	to cross one's fingers
decir tu domicilio	to state one's address
derribar una botella	to knock over a bottle
doblar un alambre	to bend a wire
enrollar un ovillo	to wind a ball of yarn
escuchar el reloj	to listen to the clock
fruncir el ceño	to frawn
hacer un nudo	to make a knot
lanzar un beso	to blow a kiss
mirar una postal	to look at a postcard
morder la arena	to bite the sand
ponerse unas gafas	to put on a pair of glasses
pulsar un botón	to press a button
rascarse la nariz	to scratch one's nose
romper una cerilla	to break a match
sacar la lengua	to stick out one's tongue
señalar la puerta	to point to the door
soplar sobre cera	to blow on wax
tararear una melodía	to hum a melody
tomar una pastilla	to take a pill

Experiment 2

“Sitting” sentences

**Spanish sentence**	**English translation**
tocar el órgano	to play the organ
conducir un carruaje	to drive a carriage
viajar en taxi	to travel by taxi
ver la tele	to watch TV
jugar al ajedrez	to play chess
escribir una carta	to write a letter
andar en bicicleta	to ride a bicycle
coser una falda	to sew a skirt
sentarse en un taburete	to sit on a stool
esperar en una silla	to wait in a chair
montar a caballo	to ride a horse
tomar un plato de ensalada	to eat a plate of salad

“Standing” sentences

**Spanish sentence**	**English translation**
construir un tabique	to build a partition
mirar un escaparate	to watch a shop window
llamar al timbre	to ring the doorbell
pisar una araña	to step on a spider
apuntar con una escopeta	to aim with a shotgun
abrir un buzón	to open a mailbox
ordenar una estantería	to tidy up a shelf
abrazar un árbol	to hug a tree
fregar una taza	to wash a mug
colgar una percha	to hang a hanger
freír un huevo	to fry an egg
asomarse a una ventana	to peek into a window
